# NOTCH1 Activation Negatively Impacts on Chronic Lymphocytic Leukemia Outcome and Is Not Correlated to the *NOTCH1* and *IGHV* Mutational Status

**DOI:** 10.3389/fonc.2021.668573

**Published:** 2021-05-26

**Authors:** Stefano Baldoni, Beatrice Del Papa, Filomena De Falco, Erica Dorillo, Carlo Sorrentino, Chiara Rompietti, Francesco Maria Adamo, Manuel Nogarotto, Debora Cecchini, Elena Mondani, Estevao Carlos Silva Barcelos, Lorenzo Moretti, Maria Grazia Mameli, Bianca Fabi, Daniele Sorcini, Arianna Stella, Raffaella Giancola, Francesco Guardalupi, Francesca Ulbar, Sara Plebani, Valerio Guarente, Emanuela Rosati, Marta Di Nicola, Michele Marchioni, Mauro Di Ianni, Paolo Sportoletti

**Affiliations:** ^1^ Institute of Hematology-Centro di Ricerca Emato-Oncologica (CREO), Department of Medicine and Surgery, University of Perugia, Perugia, Italy; ^2^ Department of Medicine and Aging Sciences, “G. d’Annunzio” University of Chieti-Pescara, Chieti, Italy; ^3^ Department of Biological Sciences, Postgraduate Program in Biotechnology (UFES), Federal University of Espirito Santo, Vitoria, Brazil; ^4^ Department of Oncology and Hematology, “Santo Spirito” Hospital, Pescara, Italy; ^5^ Hematology Unit, “San Salvatore” Hospital, L’Aquila, Italy; ^6^ Department of Medicine and Surgery, University of Perugia, Perugia, Italy; ^7^ Department of Medical, Oral and Biotechnological Sciences, “G. d’Annunzio” University of Chieti-Pescara, Chieti, Italy

**Keywords:** risk stratification, NOTCH1 activation, chronic lymphocytic leukemia, *IGHV* mutation, *NOTCH1* mutation

## Abstract

NOTCH1 mutations and deregulated signal have been commonly found in chronic lymphocytic leukemia (CLL) patients. Whereas the impact of *NOTCH1* mutations on clinical course of CLL has been widely studied, the prognostic role of NOTCH1 activation in CLL remains to be defined. Here, we analyzed the activation of NOTCH1/NOTCH2 (ICN1/ICN2) and the expression of JAGGED1 (JAG1) in 163 CLL patients and evaluated their impact on TTFT (Time To First Treatment) and OS (Overall Survival). NOTCH1 activation (ICN1+) was found in 120/163 (73.6%) patients. Among them, 63 (52.5%) were *NOTCH1* mutated (ICN1+/mutated) and 57 (47.5%) were *NOTCH1* wild type (ICN1+/WT). ICN1+ patients had a significant reduction of TTFT compared to ICN1-negative (ICN1−). In the absence of *NOTCH1* mutations, we found that the ICN1+/WT group had a significantly reduced TTFT compared to ICN1− patients. The analysis of *IGHV* mutational status showed that the distribution of the mutated/unmutated *IGHV* pattern was similar in ICN1+/WT and ICN1− patients. Additionally, TTFT was significantly reduced in ICN1+/ICN2+ and ICN1+/JAG1+ patients compared to ICN1−/ICN2− and ICN1−/JAG1− groups. Our data revealed for the first time that *NOTCH1* activation is a negative prognosticator in CLL and is not correlated to *NOTCH1* and *IGHV* mutational status. Activation of NOTCH2 and JAGGED1 expression might also influence clinical outcomes in this group, indicating the need for further dedicated studies. The evaluation of different NOTCH network components might represent a new approach to refine CLL risk stratification.

## Introduction

*NOTCH1* mutations and deregulated signal have been commonly found in chronic lymphocytic leukemia (CLL) patients (1, 2). Clonal *NOTCH1* mutations have been detected in up to 20% of CLL while recent evidence showed that the NOTCH1 pathway can be constitutively activated independently of mutation in about 50% of patients (3, 4).

However, whereas the impact of *NOTCH1* mutations on the clinical course of CLL has been widely studied (5, 6), the role of NOTCH1 activation remains to be defined. Furthermore, we previously showed that CLL cells exhibit a constitutively activated NOTCH2 and express the JAGGED1 ligand which is involved in IL4-induced CLL cell survival (2, 7–9) whose prognostic role in CLL is largely unknown. A better understanding of the NOTCH network in CLL may not only help to refine prognosis, but also expand therapeutic strategies based on the use of single anti-NOTCH molecules (10) or their combination with new drugs (11).

In the present retrospective study, we analyzed the role of NOTCH1/NOTCH2 activation and JAGGED1 expression in the outcome of CLL patients and weighed up their impact in comparison with *NOTCH1* and *IGHV* mutational status.

## Materials and Methods

Neoplastic B cells were obtained from the blood of patients at the diagnosis of CLL using Ficoll density-gradient centrifugation followed by sheep erythrocyte rosetting. The purity of CD19+/CD5+ cells (90%, range 70–99%) was determined by flow cytometry (EPICS-XLMCL; Beckman Coulter, Brea, CA, USA) analysis using anti-CD45, CD19, CD5, CD11b, CD3 monoclonal antibodies (moAbs) on 7AAD negative (Beckman Coulter, Brea, CA, USA).

Western blot (WB) analysis was performed on protein lysates (20 µg) extracted from CLL cells freshly isolated from peripheral blood samples collected at diagnosis, using the Cleaved NOTCH1 Val1744 (clone D3B8) moAb (Cell Signaling Technology, Beverly, MA, USA) to detect activated NOTCH1 intracellular domain (ICN1+), the polyclonal NOTCH2 antibody Cleaved-Val1697 (Sigma-Aldrich, St. Louis, MO, USA), anti-JAGGED1 C-terminal (clone TS1.15H) (DSHB Developmental Studies Hybridoma Bank, IA, USA) and anti-GAPDH moAb (Sigma-Aldrich, St. Louis, MO, USA). Signals were detected using appropriate horseradish peroxidase-conjugated secondary antibodies. Densitometric analysis was performed using Quantity One software (Bio-Rad, Hercules, CA, USA). GAPDH was used as an analysis control. Samples in our cohort were classified as ICN1 negative (ICN1−) when no signal was detected after 30 min exposure (long exposure), while all the others were classified as positive. To exclude technical issues causing false ICN1−, in each WB session, we evaluated the quality of ICN1 staining, loading Molt4 cell line lysate as positive control for delCT mutation, or Jurkat cell line lysate as positive control for NOTCH1 activation.

Genomic DNA was isolated using the Maxwell^®^ system (Promega Corporation, Madison, WI, USA) and the c.7544–7545 delCT* NOTCH1* mutation screening was performed using droplet digital PCR (ddPCR) to determine the percentage of allelic burden. The droplet generated included ddPCR Supermix for Probes2× (no dUTP; Bio-Rad), NOTCH1 probes assays 20× (FAM probe dHsaCP2500500 and HEX Probe dHsaCP2500501; Bio-Rad) and 150 ng of sample DNA. The mix was amplified by PCR according to the probes’ data sheet and analyzed by QX200 Droplet Reader (Bio-Rad). Scatter plot analysis specifically determine the *NOTCH1* allelic frequency mutation. The false positive threshold was determined as the upper limit of the mutant allele concentration error bars of the WT control, whereas the value of 0.03% was defined as the lower limit for the *NOTCH1* mutation. Patients with three positive events, out of a total of 10,000, were defined as *NOTCH1* mutated. The value of 20% define the subclonal *vs* clonal groups.

*IGHV* mutational status was analyzed according to ERIC recommendations.

Statistical analyses were performed with Prism Software (GraphPad Software, San Diego, CA, USA). Clinical and biological features between groups were compared using the Fisher’s exact test for categorical data and the non-parametric Mann–Whitney for non-paired data. Survival was calculated from the date of diagnosis to the date of first treatment (TTFT) or date of death (OS) using the Kaplan–Meier method. A multivariable Cox’s regression model was fitted to estimate hazard ratios (HRs) and their 95% confidence intervals (95%CIs), using the R software environment (version 4.0.3). A *p* value < 0.05 was considered significant.

The study was conducted according to the guidelines of the Declaration of Helsinki and approved by the local Ethics Committee of the University of Perugia, Perugia, Italy (approval 2015–001).

## Results and Discussion

Peripheral blood samples were obtained from CLL patients at diagnosis after informed consent. Patient characteristics are described in **Supplementary Table 1**.

**Figure 1:**
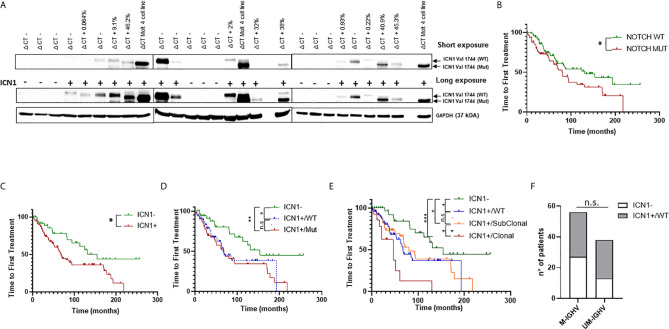
Analysis of NOTCH1 activation and mutations in CLL patients. **(A)** Western blot analysis of NOTCH1 activation in whole cell lysates from 163 primary CLL cells. Short and long exposure of NOTCH1 activation (ICN1−/+) in 24 representative *NOTCH1* WT (ΔCT−) and *NOTCH1* mutated (ΔCT+, with relative AFs) CLL cases. ICN1 Val 1744 (WT) indicates the ICN1 *NOTCH1* WT band, whereas ICN1 Val 1744 (Mut) indicates the ICN1 *NOTCH1* mutated band (the difference in molecular weight is due to the presence of the deletion of a portion of PEST1). The Molt4 cell line was used as positive control. Protein loading was assessed by reprobing the blots with an anti-GAPDH antibody. **(B)** Kaplan–Meier analysis was used to determine TTFT in *NOTCH1* mutated patients (*NOTCH1* MUT) (n = 70) compared to *NOTCH*1 WT patients (n = 93) (80 *vs* 131 months, *p* = 0.04). Kaplan–Meier analysis was used to determine the influence on TTFT of the NOTCH1 activation and mutation status. Time to first treatment analysis according to: **(C)** NOTCH1 activation status (ICN1−, n = 43 and ICN1+, n = 120); **(D)** NOTCH1 activation status (ICN1−, n = 43) and *NOTCH1* mutational status: ICN1+/WT (n = 57) and ICN1+/Mut (n = 63); **(E)** NOTCH1 activation status (ICN1−, n = 43) and *NOTCH1* mutational status: ICN1+/WT (n = 57), ICN1+/Clonal mutated (n = 16) and ICN1+/Subclonal mutated (n = 47). **(F)** Stacked bar graph shows the *IGHV* mutational status in CLL patients with ICN1− compared to ICN1+/WT (Fisher’s exact Test). ***p < 0.001; **p < 0.01; *p < 0.05; and n.s., not significant.

To identify NOTCH1 activated CLL cases, we performed Western blot (WB) analysis of the cleaved (Val1744) intracellular domain of NOTCH1 (ICN1) in 163 patients, which revealed ICN1 expression in 120 samples (73.6%) (**Supplementary Table 1** and **Figure 1A**). Among ICN1+ patients, 63/120 (52.5%) were *NOTCH1* mutated (ICN1+/mutated) while 57/120 (47.5%) were *NOTCH1* WT (ICN1+/WT).

Patients were defined mutated or WT based on the *NOTCH1* allelic frequency (AF) assigned by droplet digital PCR (ddPCR): mutated patients had an AF >0.03%, while WT patients had an AF ≤0.03%. Furthermore, *NOTCH1* mutations were classified as clonal (AF >20%) or subclonal (AF <20%) (**Supplementary Figure 1**). *NOTCH1* subclonal mutations (AF =0.6%) were found only in seven ICN1− patients out of 43 and were not associated with activation of NOTCH1 signaling, as demonstrated by the absence of ICN1 in WB analysis, even using a long exposure. These data suggest that the activation of the NOTCH1 pathway is not strictly dependent on the presence of mutations.

Subsequently, we analyzed the TTFT of patients bearing *NOTCH1* mutations and we found that it was significantly shorter if compared to the TTFT of patients bearing the WT allele (80 *vs* 131 months, *p* = 0.04) (**Figure 1B**). This confirmed the role of the *NOTCH1* mutation as a negative prognostic factor in CLL, as reported by Minervini et al., 2016 (3).

Then, we assessed the prognostic significance of ICN1 activation on TTFT and OS, and we found that ICN1+ patients (including both *NOTCH1* mutated and WT) had a significant reduction of TTFT compared to ICN1− (*p* < 0.05; **Figure 1C**). OS was not significantly different (**Supplementary Figure 2A**). Analysis of ICN1+/mutated *versus* ICN1+/WT patients showed a similar TTFT (**Figure 1D**), as well as ICN1−/mutated *versus* ICN1−/WT patients (*p* = 0.9; data not shown), suggesting that NOTCH1 activation could impact on TTFT regardless of the *NOTCH1* mutation. Surprisingly, we found that the ICN1+/WT group had a significantly reduced TTFT compared to ICN1− patients (67 *vs* 154 months; *p* < 0.05) (**Figure 1D**). OS was similar in both groups (**Supplementary Figure 2B**). Furthermore, within ICN1+ patients, when we considered clonal *vs*. subclonal *NOTCH1* mutations, TTFT was significantly reduced in patients carrying a clonal mutations (**Figure 1E**) suggesting that allelic ratio remains a key factor.

These data suggested that NOTCH1 activation was a negative prognosticator in CLL, regardless the *NOTCH1* mutation status. These findings were further strengthened by recent lines of evidence that demonstrated how NOTCH1 signaling is able to induce equivalent transcriptional programs in *NOTCH1* mutated and *NOTCH1* WT cases (4).

To further explore the prognostic role of NOTCH1 activation in *NOTCH1* WT patients, we analyzed its correlation with the *IGHV* mutational status. Results showed that the distribution of the mutated/unmutated *IGHV* pattern was similar in ICN1+/WT and ICN1− patients (**Figure 1F**). Given the poor outcome of ICN1+/WT patients, non-mutated but active NOTCH1 might unveil a new prognostic category with a poor outcome which is not correlated to the *IGHV* mutational status.

To confirm the role of NOTCH1 activation as an independent prognostic marker in CLL, we performed a multivariate Cox regression analysis, including *NOTCH1* mutational status, *IGHV* status, sex, Rai stage (0–1) and FISH results. Surprisingly, NOTCH1 activation (ICN1+) failed to be significant, if considered alone (**Supplementary Table 2**). However, if combined (ICN1+/Mut) with *NOTCH1* mutational status, which also failed to be an independent prognostic marker when considered alone (3), NOTCH1 activation revealed an independent prognostic effect on the outcome of interest, even after adjustment for *IGHV* mutational status and other confounders prognosticator (HR: 2.14, *p* = 0.046) (**Supplementary Table 2**). Separating clonal and subclonal mutations, this prognostic effect is lost in ICN1+/Subclonal patients while it is maintained in ICN1+/Clonal patients, confirming what had been demonstrated by the univariate analysis (**Figure 1E**).

**Figure 2:**
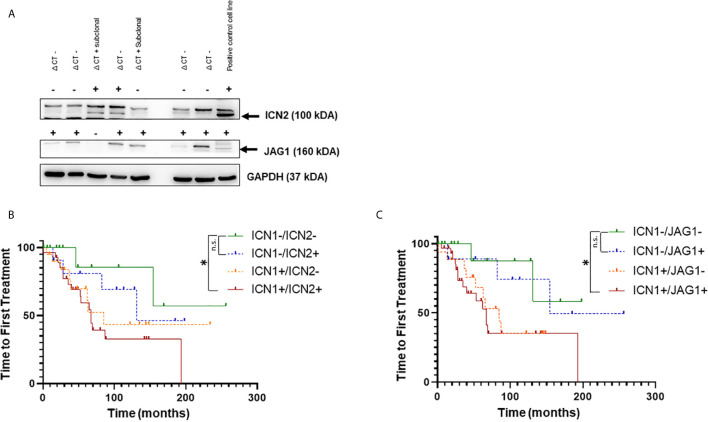
Analysis of NOTCH1, NOTCH2 activation and JAGGED1 expression in CLL patients. **(A)** Representative Western blot analysis of NOTCH2 activation and JAGGED1 expression in whole cell lysates from 130 primary CLL cells. Protein loading was assessed by reprobing the blots with an anti-GAPDH antibody. Kaplan–Meier analysis was used to determine the influence on TTFT of the NOTCH2 activation status and JAGGED1 expression in 125 CLL patients. Time to first treatment analysis according to: **(B)** activation status of NOTCH1 (n = 49) compared to NOTCH2 (n = 41) activation status in *NOTCH1* WT patients; **(C)** NOTCH1 activation status (n = 50) compared to JAGGED1 expression (n = 40) in *NOTCH1* WT patients. *p < 0.05; and n.s., not significant.

To investigate the role of NOTCH2 activation and JAG1 expression in CLL progression, we performed WB analysis of NOTCH2 (ICN2*)* and JAG1 expression in 130 patients (**Figure 2A**) and analyzed their impact on TTFT and OS.

First, to confirm that, also in this group of patients, NOTCH1 activation was a negative prognosticator, we performed WB analysis of ICN1 in 125 patients among the 130 patients in the cohort. The results demonstrated that there was a significant reduction in TTFT between ICN1− and ICN1+ patients (**Supplementary Figure 3A**) (154 *vs*. 71 months *p* = 0.041). Furthermore, ICN1− patients had significantly longer TTFT than ICN1+/WT patients (154 *vs*. 67; *p* < 0.05) and ICN1+/mutated patients (154 *vs*. 71; *p* < 0.05), while TTFT was similar between ICN1+/WT patients and ICN1+/mutated patients (67 *vs*. 71; *p* > 0.05), confirming the role of NOTCH1 activation as prognostic marker, not correlated to *NOTCH1* mutation (**Supplementary Figure 3B**).

The ICN2 protein was positive in 71/130 (55%) while JAG1 in 69/130 (53.1%) samples. Since the prognostic impact of the *NOTCH1* mutation is now well known, samples with *NOTCH1* mutation were excluded from the analysis to better define the role of the activation state of ICN1, ICN2 and JAG1 expression. Furthermore, the evaluation of the prognostic impact of ICN2 and JAG1 was performed in patients stratified for the presence or the absence of ICN1.

The expression of ICN2 (n = 12) or JAG1 (n = 10) alone showed a better TTFT (**Figures 2B, C**) than activated ICN1 only (n = 20) (131 and 154 *vs* 85 months; *p* = 0.49; *p* = 0.17, respectively).

Interestingly, TTFT was significantly reduced in ICN1+/ICN2+ and ICN1+/JAG1+ samples compared to ICN1−/ICN2− and ICN1−/JAG1− (**Figures 2B, C**; *p* < 0.05). Expression of both ICN1 and ICN2 or JAG1 did not affect OS (**Supplementary Figures 2C, D**). Altogether, these results are not able to support an additional detrimental effect of ICN2 and JAG1 on CLL outcome when leukemic cells co-express or not ICN1. To clarify this point, further studies on larger cohorts of patients are needed.

Analysis of the concomitant presence of ICN1, ICN2 and JAG1 proteins in 125 patients revealed 9.6% (n = 12) triple negative (ICN1−/ICN2−/JAG1−) and 28% (n = 35) triple positive (ICN1+/ICN2+/JAG1+) CLL (**Supplementary Figure 4A**). Interestingly, this latter group showed a tendency toward a reduced TTFT compared to triple negative CLL, in the absence of *NOTCH1* mutations (**Supplementary Figure 4B**).

Although not significant for the small number of patients analyzed, these data underlie the importance of NOTCH network profiling in CLL.

Altogether, our data provide insights into the prognostic role of NOTCH1 activation in CLL lacking *NOTCH1* mutation. Notably, we have identified ICN1+CLL as a new group of patients with a negative outcome, not correlated to *NOTCH1* and *IGHV* mutational status.

The aberrant NOTCH1 signaling in CLL cells regulates several genes that influence key biological aspects of neoplastic cells, such as apoptosis, cell growth, cell migration, interactions with the microenvironment and B Cell Receptor (BCR) activation. It has been shown that a synergistic cooperation between NOTCH1 and BCR strongly supports the survival/proliferation of CLL cells and contributes to a progression of the disease (11) but also to the transformation into Richter’s syndrome (RS) (12). Furthermore, it has been recently shown that ligand-dependent activation of NOTCH1 signaling, *via* constitutive PI3K/AKT activation, promotes CLL transformation towards RS in Eμ-*TCL1* mice *in vivo* (13), thus further supporting the association between non-mutational NOTCH1 activation and a poor prognosis. Based on these notions, the most likely hypothesis is that, in CLL cells, *NOTCH1* mutations, by stabilizing ICN1 levels, potentiate an oncogenic signaling that was initiated by interactions between the NOTCH1 receptor expressed on leukemic cells and ligands expressed on cellular microenvironment (14). Previous data have shown that NOTCH1 activation promotes a genetic program in CLL cells similar to that induced by the *NOTCH1* mutation (4) and our data show, for the first time, a correlation between these transcriptional levels and clinical outcome.

We have also shown that activation of NOTCH2 and JAGGED1 expression might also influence clinical outcomes in ICN1+CLL patients, indicating the need for further dedicated studies. Overall, our data implicate the evaluation of different NOTCH network components as a new approach to refine CLL risk stratification.

However, given the limited size of our cohort, further studies in a larger cohort of patients are needed to confirm the significance of non-mutational NOTCH1 activation and different NOTCH network components in CLL prognosis.

## Data Availability Statement

The raw data supporting the conclusions of this article will be made available by the authors, without undue reservation.

## Ethics Statement

The study was conducted according to the guidelines of the Declaration of Helsinki and approved by the local Ethics Committee of the University of Perugia, Perugia, Italy (approval 2015–001). The patients/participants provided their written informed consent to participate in this study.

## Author Contributions

Conception and design: SB, BDP, and PS. Development of methodology: SB, BDP, FDF, ED, CR, MN, EM, BF, VG, ER, and PS. Acquisition of data (acquired and managed patients, provided facilities, etc.): VG and PS. Analysis and interpretation of data (e.g., statistical analysis, biostatistics, computational analysis): SB, BDP, FDF, ED, CR, EM, MN, FMA, ECSB, VG, ER, MDI, MDN, MM, and PS. Writing, review, and/or revision of the manuscript: SB, BDP, MDI, ER, CS, and PS. Administrative, technical, or material support (i.e., reporting or organizing data, constructing databases): SB, BP, FDF, ED, MN, ECSB, FMA, LM, MGM, DS, AS, RG, FG, FU, SP, and PS. Study supervision: PS. All authors contributed to the article and approved the submitted version.

## Funding

This research was funded by the Associazione Italiana per la Ricerca sul Cancro (AIRC) (MFAG 2015-ID.17442 Project, and IG 2018–ID. 21352 Project to PS); FIRC-AIRC (three-year fellowship “Filomena Todini” ID. 23928 to CR), Italian Ministry of Education, University and Research (MIUR) (Scientific Independence of Young Researchers Project-ID. RBSI14GPBL to PS); Italian Ministry of Health (Ricerca Finalizzata, RF-2016-02364383 to MDI); Gilead Fellowship Program 2016 (to PS) and Associazione Italiana contro le Leucemie-Linfomi e Mieloma (AIL), L’Aquila Section, Italy.

## Conflict of Interest

The authors declare that the research was conducted in the absence of any commercial or financial relationships that could be construed as a potential conflict of interest.

## Supplementary Material

Supplementary Table 1 Clinical and biological characteristics of the CLL patients analyzed.

Supplementary Table 2 Multivariable Cox’s regression models included ICN1 activation and NOTCH1 mutatation status, IGHV mutational status, Sex, Rai Stage and FISH with a prognosis good (Normal and del13q14 as unique lesion), Intermediate (Trisomy 12) and poor (del 11q23 or del 17p13). ***, *p*<0.001; **, *p*<0.01; *, *p*<0.05 and *p*>0.05, not significant.

Supplementary Figure 1 Frequency distribution of the *NOTCH1* mutation in 63 CLL patients. According to the ddPCR analysis the patients were stratified in clonal and subclonal mutations.

Supplementary Figure 2 Kaplan-Meier estimates of Overall survival (OS) in CLL patients according to: **(A)** NOTCH1 activation status (ICN1-, n=43 and ICN1+, n=120). **(B)** NOTCH1 activation status (ICN1-, n=43) and *NOTCH1* mutational status: ICN1+/WT (n=57) and ICN1+/Mut (n=63). **(C)** activation status of NOTCH1 (ICN1+/-) compared to NOTCH2 activation status (ICN2+/-) in *NOTCH1* WT patients. **(D)** NOTCH1 activation status (ICN1+/-) compared to JAGGED1 expression (JAG1+/-) in *NOTCH1* WT patients.

Supplementary Figure 3 Kaplan-Meier estimates the TTFT analysis in CLL patients according to: **(A)** NOTCH1 activation status (ICN1-, n=36 and ICN1+, n=89). **(B)** NOTCH1 activation status (ICN1-, n=36) and *NOTCH1* mutational status: ICN1+/WT (n=48) and ICN1+/Mut (n=41). *, *p*<0.05 and n.s., not significant.

Supplementary Figure 4 NOTCH1/NOTCH2 activation and JAGGED1 expression and TTFT. **(A)** Heat map of ICN1, ICN2 and JAG1 status in 125 patients with CLL revealed by Western blot analysis. Kaplan-Meier to evaluate TTFT in CLL patients according **(B)** to triple negative (ICN1-/ICN2-/JAG1-) and triple positive (ICN1+/ICN2+/JAG1+) status.
