# Supplementary material for: NOTCH1 Activation Negatively Impacts on Chronic Lymphocytic Leukemia Outcome and Is Not Correlated to the NOTCH1 and IGHV Mutational Status
Source: Front Oncol. 2021 May 26;11:668573. doi: 10.3389/fonc.2021.668573 (PMC8187905; doi:10.3389/fonc.2021.668573)
Supplement: Supplementary Table 2 — Multivariable Cox’s regression models included ICN1 activation and NOTCH1 mutatation status, IGHV mutational status, Sex, Rai Stage and FISH with a prognosis good (Normal and del13q14 as unique lesion), Intermediate (Trisomy 12) and poor (del 11q23 or del 17p13). ***, p<0.001; **, p<0.01; *, p<0.05 and p>0.05, not significant. [file Table_2.docx]

**SUPPLEMENTARY TABLE 2****.** Multivariable Cox’s regression models.

| **Multivariable Cox’s Model** | **Levels** | **Hazard Ratio (multivariable)** |
| --- | --- | --- |
| *ICN1 Status* | ICN1- | - |
|  | ICN1+/WT | 1.50 (0.70-3.18, p=0.297) |
|  | ICN1+/Mut | 2.14 (1.01-4.51, p=0.046) |
|  | ICN1+/Subclonal Mut | 1.66 (0.76-3.64, p=0.205) |
|  | ICN1+/Clonal Mut | 6.57 (2.38-18.11, p<0.001) |
| *IGHV* | Mutated | - |
|  | Unmutated | 4.92 (2.61-9.27, p<0.001) |
| *Sex* | Female | - |
|  | Male | 1.05 (0.57-1.92, p=0.872) |
| *Rai Stage* | Stage 0-I | - |
|  | Stage II-IV | 2.08 (1.22-3.55, p=0.007) |
| *FISH Prognosis* | Good | - |
|  | Intermediate | 0.66 (0.29-1.52, p=0.333) |
|  | Poor | 2.23 (1.19-4.19, p=0.012) |
