# Supplementary figures and images for: NOTCH1 Activation Negatively Impacts on Chronic Lymphocytic Leukemia Outcome and Is Not Correlated to the NOTCH1 and IGHV Mutational Status
Source: Front Oncol. 2021 May 26;11:668573. doi: 10.3389/fonc.2021.668573 (PMC8187905; doi:10.3389/fonc.2021.668573)

## Slide 1
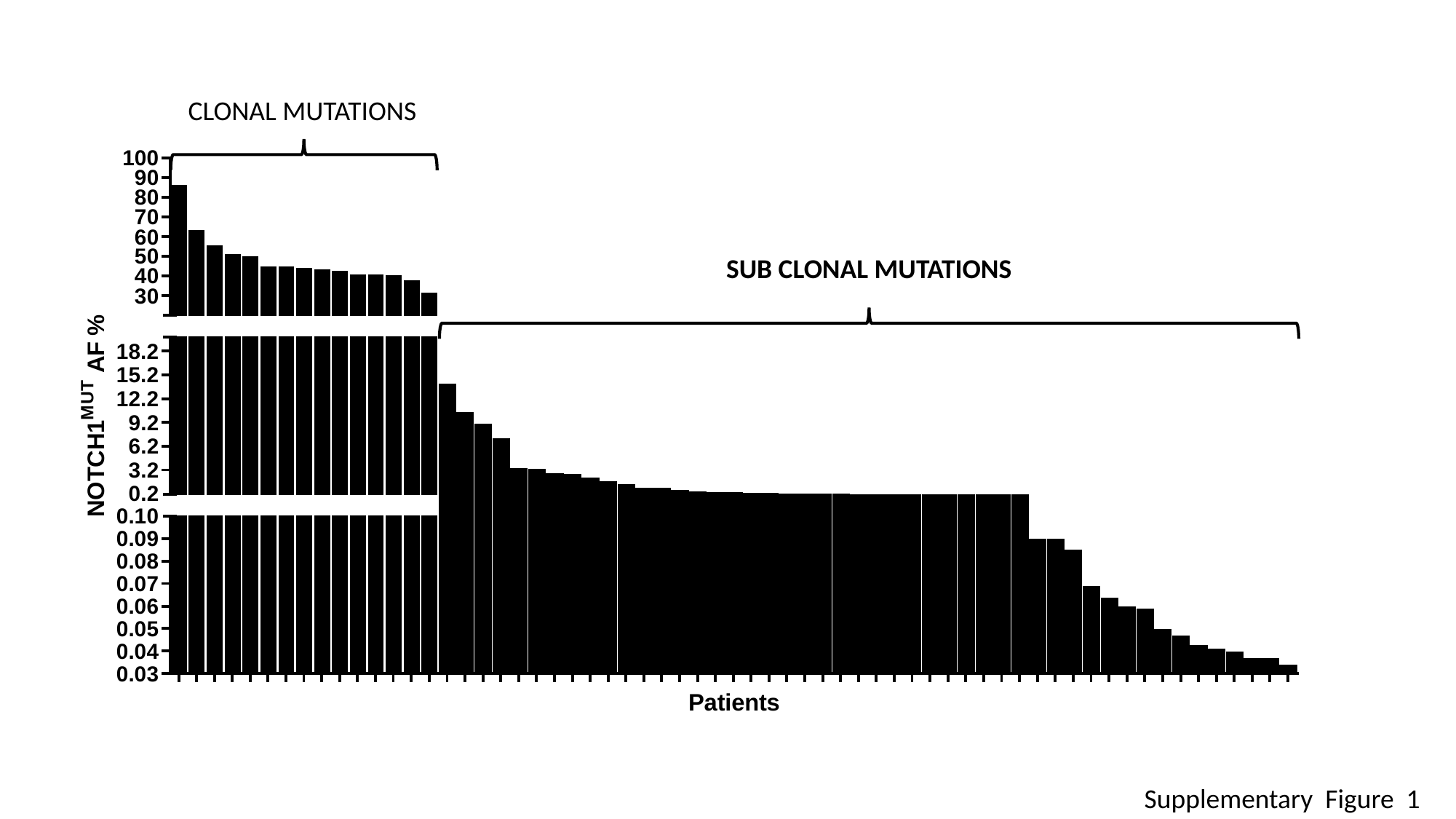

CLONAL MUTATIONS
SUB CLONAL MUTATIONS
Supplementary Figure 1

Supplement: Supplementary Figure 1 — Frequency distribution of the NOTCH1 mutation in 63 CLL patients. According to the ddPCR analysis the patients were stratified in clonal and subclonal mutations. [file Presentation_1.pptx]
