# Supplementary material for: NOTCH1 Activation Negatively Impacts on Chronic Lymphocytic Leukemia Outcome and Is Not Correlated to the NOTCH1 and IGHV Mutational Status
Source: Front Oncol. 2021 May 26;11:668573. doi: 10.3389/fonc.2021.668573 (PMC8187905; doi:10.3389/fonc.2021.668573)
Supplement: Supplementary Figure 2 — Kaplan-Meier estimates of Overall survival (OS) in CLL patients according to: (A) NOTCH1 activation status (ICN1-, n=43 and ICN1+, n=120). (B) NOTCH1 activation status (ICN1-, n=43) and NOTCH1 mutational status: ICN1+/WT (n=57) and ICN1+/Mut (n=63). (C) activation status of NOTCH1 (ICN1+/-) compared to NOTCH2 activation status (ICN2+/-) in NOTCH1 WT patients. (D) NOTCH1 activation status (ICN1+/-) compared to JAGGED1 expression (JAG1+/-) in NOTCH1 WT patients. [file Presentation_2.pptx]

## Slide 1
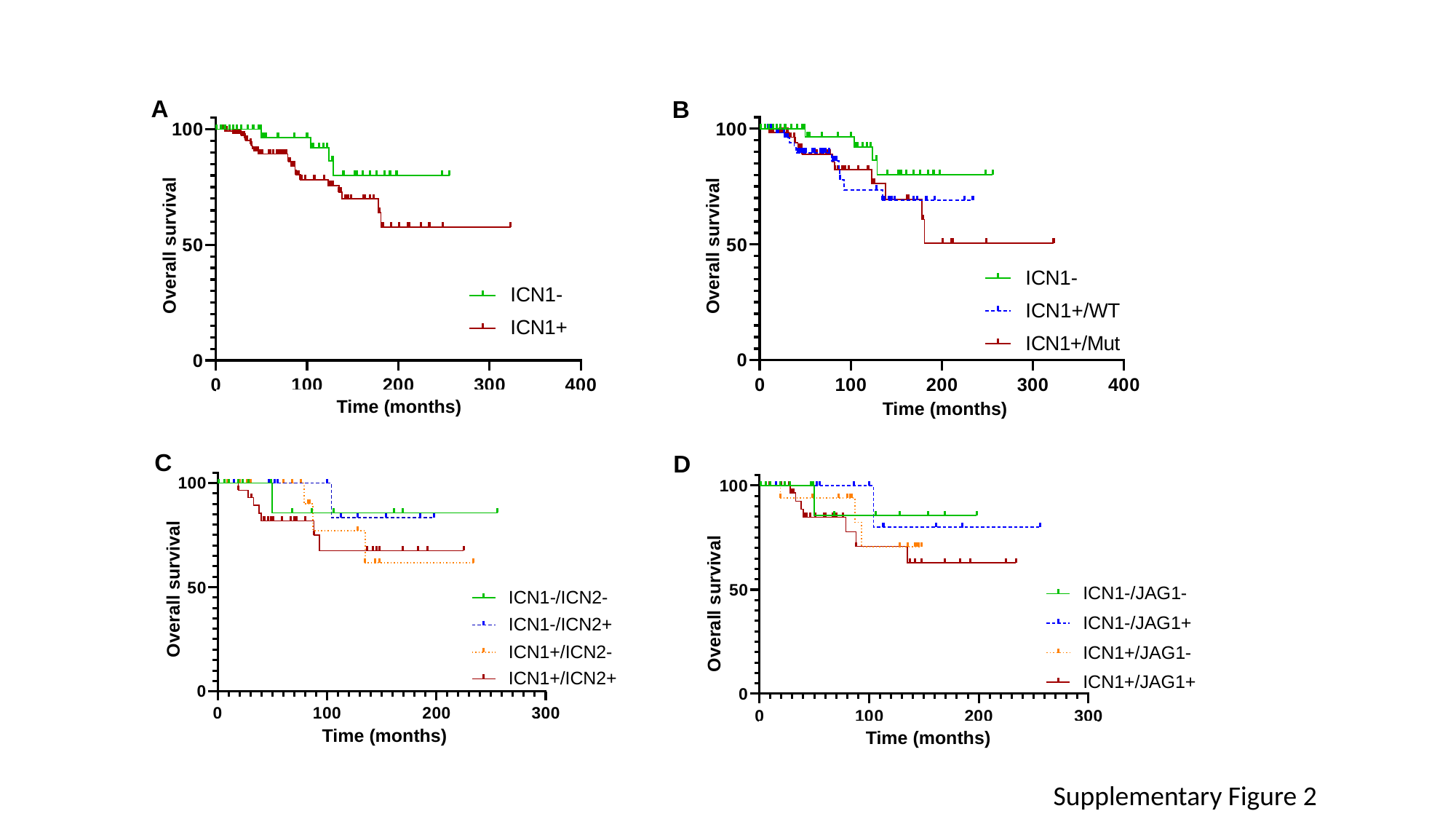

Overall survival
Time (months)
A
B
Overall survival
Time (months)
Overall survival
Time (months)
C
D
Overall survival
Time (months)
Supplementary Figure 2
