# Supplementary material for: NOTCH1 Activation Negatively Impacts on Chronic Lymphocytic Leukemia Outcome and Is Not Correlated to the NOTCH1 and IGHV Mutational Status
Source: Front Oncol. 2021 May 26;11:668573. doi: 10.3389/fonc.2021.668573 (PMC8187905; doi:10.3389/fonc.2021.668573)
Supplement: Supplementary Figure 3 — Kaplan-Meier estimates the TTFT analysis in CLL patients according to: (A) NOTCH1 activation status (ICN1-, n=36 and ICN1+, n=89). (B) NOTCH1 activation status (ICN1-, n=36) and NOTCH1 mutational status: ICN1+/WT (n=48) and ICN1+/Mut (n=41). *, p<0.05 and n.s., not significant. [file Presentation_3.pptx]

## Slide 1
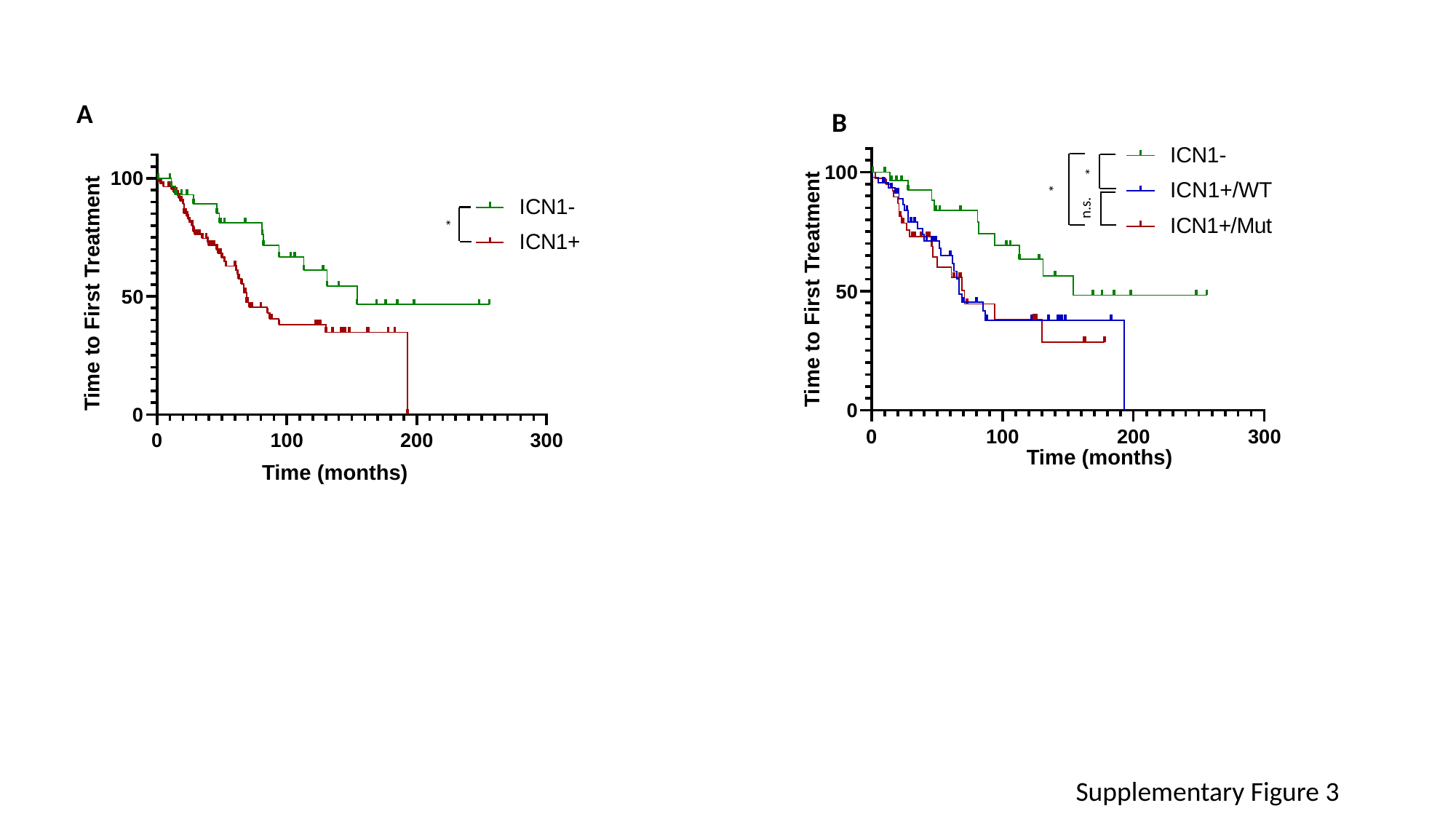

A
B
*
*
n.s.
Time to First Treatment
Time (months)
*
Time to First Treatment
Time (months)
Supplementary Figure 3
