# Supplementary material for: NOTCH1 Activation Negatively Impacts on Chronic Lymphocytic Leukemia Outcome and Is Not Correlated to the NOTCH1 and IGHV Mutational Status
Source: Front Oncol. 2021 May 26;11:668573. doi: 10.3389/fonc.2021.668573 (PMC8187905; doi:10.3389/fonc.2021.668573)
Supplement: Supplementary Figure 4 — NOTCH1/NOTCH2 activation and JAGGED1 expression and TTFT. (A) Heat map of ICN1, ICN2 and JAG1 status in 125 patients with CLL revealed by Western blot analysis. Kaplan-Meier to evaluate TTFT in CLL patients according (B) to triple negative (ICN1-/ICN2-/JAG1-) and triple positive (ICN1+/ICN2+/JAG1+) status. [file Presentation_4.pptx]

## Slide 1
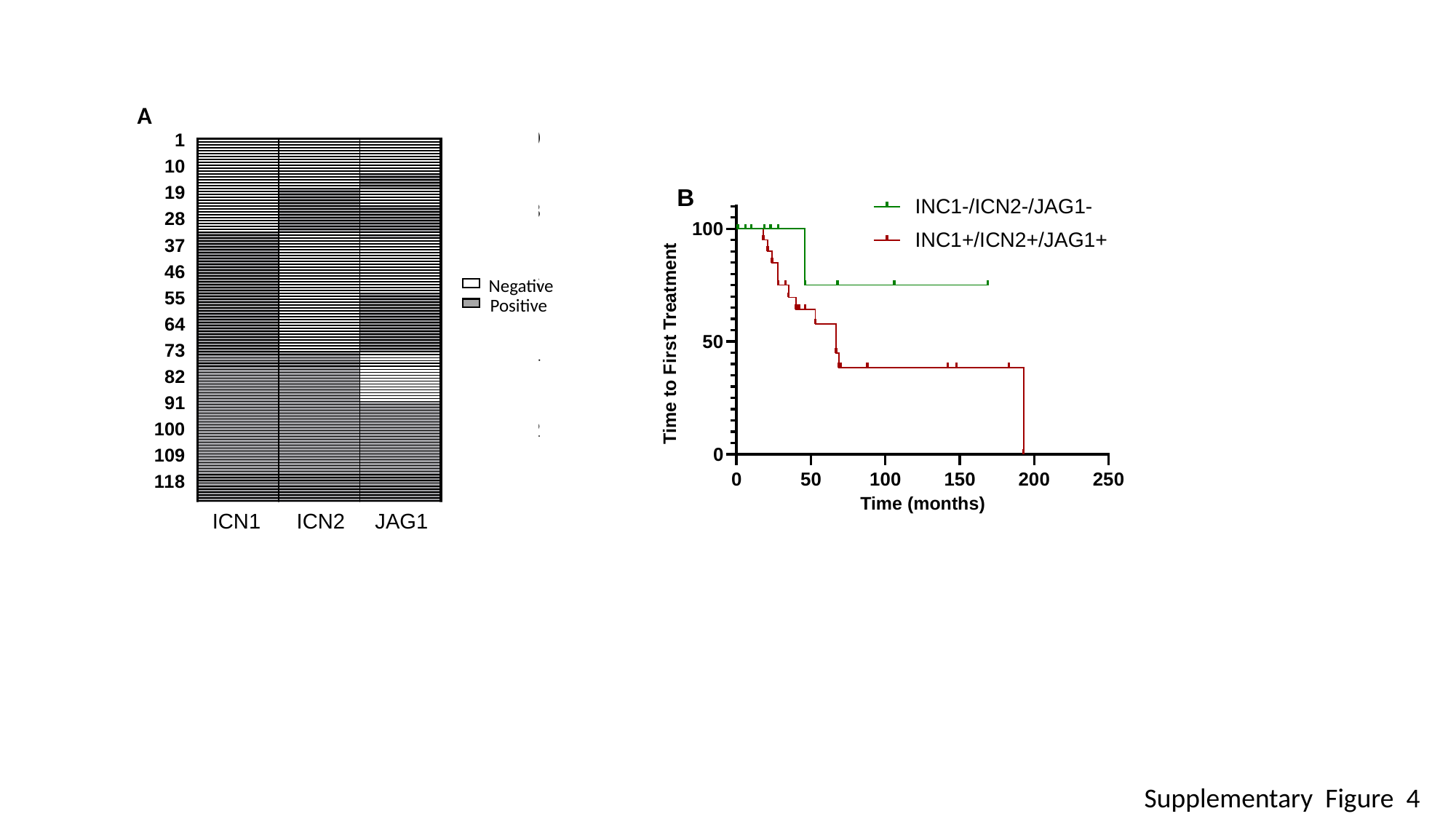

A
ICN1 ICN2 JAG1
Time to First Treatment
Time (months)
B
Negative
Positive
Supplementary Figure 4
